# Rigler’s Triad Another Challenge to Remember

**DOI:** 10.31662/jmaj.2025-0075

**Published:** 2025-05-26

**Authors:** Vitorino Modesto dos Santos, Kin Modesto Sugai

**Affiliations:** 1 Department of Medicine, Armed Forces Hospital, Brasilia-DF, Brazil; 2 Catholic University of Brasília-DF, Brasilia, Brazil; 3 Postgraduate Course of Management, Technology, and Information Security, University of Brasília, Brasília, Brazil

**Keywords:** Cholelithiasis, Diagnosis, Rigler’s Triad, Treatment

Dear Editor,

Our interest in the Rigler triad (RT) increased after reading the illustrative case report recently published in this journal by Ono and Kitagawa ^(1)^. These authors highlighted the antecedent of cholelithiasis and cholecystitis, besides the development of a biliodigestive fistula with the occurrence of a gallstone ileal obstruction; the main imaging tips were pneumobilia, small-bowel obstruction, and ectopic gallstone ^(1)^. The 100-year-old woman underwent successful surgical treatment by laparotomy; the authors emphasized the role of cholecystoduodenal or cholecystogastric fistulas in RT ^(1)^. In this setting, the following comments may reinforce the aim of the first referenced study. Alhomaid et al. ^(2)^ reported an 80-year-old woman with mild abdominal pain, pneumobilia, and a gallstone impacted in the ileum; diagnosed with RT, the patient underwent a robotic exploration plus enterotomy followed by an uneventful postoperative period. The diagnosis was favored by reviewing previous images of the stone in the gallbladder; next, the authors highlighted the role of unified electronic medical record systems to obtain early diagnoses of RT in older patients by enabling prompt access to old imaging studies ^(2)^. González-Robles et al. ^(3)^ described a 94-year-old woman with symptoms of acute intestinal obstruction, and the abdominal images confirmed the diagnosis of RT. The patient underwent an exploratory laparotomy with enterotomy to remove 3 gallstones from the ileum and evolved with an unremarkable immediate postoperative outcome ^(3)^. The authors emphasized the inclusion of RT in the differential diagnosis of acute intestinal obstruction, primarily in older people, and prompt surgical intervention ^(3)^. Qureshi and Sabala reported a 74-year-old woman who had pneumobilia and ileal obstruction caused by a non-calcified gallstone not revealed in the abdominal images; RT was then confirmed by laparotomy, with the removal of the obstructing gallstone ^(4)^. Zabeirou et al. ^(5)^ described an 83-year-old woman with antecedent of an asymptomatic large gallstone, who had an acute ileal obstruction and confirmed RT. She underwent a laparotomy, and a giant gallstone was obstructing the distal ileum; the enterolithotomy was promptly performed with an uneventful postoperative period ^(5)^.

A total of 5 patients were reviewed (Table 1), all women; the average age was 74-100 years; all cases had surgical intervention (laparotomy: 80%), and all outcomes were favorable. Reporting challenging entities may contribute to reducing misdiagnoses.

**Table 1.** Data of 17 Patients with Diagnoses of Rigler’s Triad Published in 2024 and 2025.

References	Ages	Gender	Obstruction	Management	Outcome
1. 2025	100 years	Female	Ilium	Laparotomy	Favorable
2. 2024	80 years	Female	Ilium	Robotic enterotomy	Favorable
3. 2024	94 years	Female	Ilium	Laparotomy	Favorable
4. 2024	74 years	Female	Ilium	Laparotomy	Favorable
5. 2024	83 years	Female	Ilium	Laparotomy	Favorable

Age range: 74-100 years; females: 100%; laparotomy: 80%; and good outcomes in all cases.

## Article Information

### Conflicts of Interest

None

### Author Contributions

Both authors substantially contributed to the concept of the work, drafted the work, critically reviewed it for important intellectual content, approved the version for publication, and agreed to be accountable for all aspects of the work to ensure that questions related to the accuracy or integrity of any part of the work were appropriately investigated and resolved.

### Approval by Institutional Review Board (IRB)

IRB approval was not required for this study.

### Informed Consent

Informed consent was not required for this study.

## References

[1] Ono R, Kitagawa I. Rigler’s triad: a radiological sign of gallstone ileus. JMA J. 2025;8(1):293-4. 39926058 10.31662/jmaj.2024-0192 PMC11799675

[2] Alhomaid A, Sarwar MZ, Jawed R, et al. From chronic gallstone to acute ileus: a case report. Cureus. 2024;16(10):e72621. 39610628 10.7759/cureus.72621 PMC11604247

[3] González-Robles ME, Menéndez-Goti LL, de Jesús González-Luna A, et al. Gallstone ileus presenting in an elderly patient: a case report. Int J Surg Case Rep. 2024;124:110440. 39405751 10.1016/j.ijscr.2024.110440 PMC11562408

[4] Qureshi H, Sabala M. Noncalcified gallstone ileus in computed tomography (CT) abdomen and pelvis with contrast. Cureus. 2024;16(9):e70524. 39479076 10.7759/cureus.70524 PMC11524646

[5] Zabeirou A, Saidou A, Younssa H, et al. An authentic radiological triad of Rigler allowing the diagnosis of gallstone ileus: a case report. Radiol Case Rep. 2024;19(4):1565-7. 38317700 10.1016/j.radcr.2024.01.022 PMC10839758

